# 2D DIGE analysis of maternal plasma for potential biomarkers of Down Syndrome

**DOI:** 10.1186/1477-5956-9-56

**Published:** 2011-09-19

**Authors:** Wendy E Heywood, Tracey E Madgett, Darrell Wang, Amanda Wallington, Julie Hogg, Kevin Mills, Neil D Avent

**Affiliations:** 1 Clinical & Molecular Genetics Unit, Institute of Child Health, University College London, 30 Guilford Street, London, WC1N 1EH, UK; 2 Centre for Research in Biomedicine, Faculty of Health and Life Sciences, University of the West of England, Frenchay Campus, Coldharbour Lane, Bristol, BS16 1QY, UK; 3 Fetal Medicine Unit, University College Hospital, London, NW1 2BU, UK; 4 Current Address: School of Biomedical and Biological Sciences, University of Plymouth, Drake Circus, Plymouth, PL4 8AA, UK

**Keywords:** 2D DIGE, Biomarkers, Down Syndrome, Maternal Plasma, Prenatal screening, Prenatal diagnosis

## Abstract

**Background:**

Prenatal screening for Down Syndrome (DS) would benefit from an increased number of biomarkers to improve sensitivity and specificity. Improving sensitivity and specificity would decrease the need for potentially risky invasive diagnostic procedures.

**Results:**

We have performed an in depth two-dimensional difference gel electrophoresis (2D DIGE) study to identify potential biomarkers. We have used maternal plasma samples obtained from first and second trimesters from mothers carrying DS affected fetuses compared with mothers carrying normal fetuses. Plasma samples were albumin/IgG depleted and expanded pH ranges of pH 4.5 - 5.5, pH 5.3 - 6.5 and pH 6 - 9 were used for two-dimensional gel electrophoresis (2DE). We found no differentially expressed proteins in the first trimester between the two groups. Significant up-regulation of ceruloplasmin, inter-alpha-trypsin inhibitor heavy chain H4, complement proteins C1s subcomponent, C4-A, C5, and C9 and kininogen 1 were detected in the second trimester in maternal plasma samples where a DS affected fetus was being carried. However, ceruloplasmin could not be confirmed as being consistently up-regulated in DS affected pregnancies by Western blotting.

**Conclusions:**

Despite the in depth 2DE approach used in this study the results underline the deficiencies of gel-based proteomics for detection of plasma biomarkers. Gel-free approaches may be more productive to increase the number of plasma biomarkers for DS for non-invasive prenatal screening and diagnosis.

## Background

Down Syndrome (or Trisomy 21, DS) is the most common aneuploidy. According to the National Down Syndrome Cytogenetic Register, UK there is an incidence of ~1 in 1600 live births in mothers below the age of 25 which rises to 1 in 40 by age 43. Current diagnosis of DS occurs via chorionic villus sampling (CVS) (11-14 weeks of gestation) or amniocentesis (15-20 weeks of gestation); both of which carry a ~1% risk of miscarriage. Screening of pregnant women also occurs using a combination of plasma protein markers, ultrasound markers and maternal age [1]. Screening markers used include pregnancy-associated plasma protein A (PAPP-A), alpha-fetoprotein (AFP) [2], human chorionic gonadotropin (hCG and its subunits), the steroid hormone unconjugated estriol (uE3) and inhibin A [3]. These biomarkers were discovered more by accident than a concerted effort to assess markers of DS in maternal plasma. They are all derived from fetal liver and placental trophoblast cells. Biochemical testing and ultrasound assessment can detect 70-96% of DS cases but with a 2.5-5% false positive rate [4-6].

Non-invasive prenatal diagnosis (NIPD) (and screening) would remove the risk of miscarriage associated with amniocentesis and CVS (see [7] for an NIPD review). With the improved technical approaches now available in the field of proteomics, there is potential to discover new panels of screening biomarkers [8]. The identification of a panel of more informative DS markers was a major goal of the European Framework VI SAFE Network of Excellence [9-15]. Proteomics, with a variety of mass spectrometric methods, has been used in several studies to define new potential biomarkers for aneuploid pregnancies [11,12,16-20]. Maternal plasma was used in half of these studies [11,12,17] and amniotic fluid in the others [16,18-20]. Five studies analysed samples from the second trimester [11,16,18-20], Kolla *et al. *[12] analysed samples from the first trimester and Nagalla *et al. *[17] analysed samples from both the first and second trimesters. A mother will be better informed, and have more choices available to her, if a potential biomarker can be used as early as possible in prenatal screening and/or diagnosis but if the expression of a biomarker is altered throughout gestation, it will give increased utility. Detection rates are higher for DS and false positive rates are lower in the first trimester than the second trimester [5,6] so the identification of new and better biomarkers for the second trimester is of paramount importance.

In the present study, 2D DIGE [21] has been used to search for potential plasma DS biomarkers. We have compared maternal plasma from women carrying either normal or DS fetuses from first and second trimesters. We have used three overlapping pH ranges (pH 4.5 - 5.5, pH 5.3 - 6.5 and pH 6 - 9) for 2DE which improves the resolution of previous studies (pH 3 - 10 [11] and pH 4 - 7 [17]). Protein identification from 2D gels was done by mass spectrometry.

## Results

### 2D DIGE Experiments

Plasma samples from pregnancies with DS and chromosomally normal fetuses were analysed using 2D DIGE. Three overlapping pH ranges were used for 2DE - pH 4.5 - 5.5, pH 5.3 - 6.5 and pH 6 - 9. Protein spots were detected, analysed and compared using Progenesis Same Spots and DeCyder v6.5 software. Statistically significant differences between expression of proteins in DS samples compared to euploid (control, Ctl) samples are shown (Table 1). Following the detection of differences, protein identifications of picked spots were determined by mass spectrometry.

**Table 1:** Proteins differentially expressed in the second trimester in the pH range 4.5 - 5.5

Spot no. (as on Figure 1)	Accession number (Swiss-Prot)	Abbreviated name	Protein name	Average fold up-regulation (DS/Ctl)	Theoretical mW (Da)	Theoretical p*I*	Scores for individual spot identifications	Number of sequenced peptides	Peptide positions within the protein	Peptides sequenced (overlap between spots identified per protein)	Coverage (%) (Total number of peptides assigned to protein)	Function
1	P00450	Ceruloplasmin (Seven spots identified for this protein)	CERU_HUMAN	3.69	122205	5.44	173, 437, 431, 460, 331, 107, 110	8, 17, 16, 15, 12, 7, 7	42 - 63 43 - 63 69 - 82 187 - 202 248 - 259 426 - 437 468 - 482 484 - 501 523 - 538 537 - 548 537 - 559 547 - 559 598 - 610 598 - 620 720 - 733 785 - 794 798 - 820 887 - 902 944 - 958	K.KLISVDTEHSNIYLQNGPDR.I K.LISVDTEHSNIYLQNGPDR.I K.ALYLQYTDETFR.T K.DIASGLIGPLIICK.K K.DNEDFQESNR.M R.EYTDASFTNR.K K.GAYPLSIEPIGVR.F K.NNEGTYYSPNYNPQSR.S K.EVGPTNADPVCLAK.M K.MYYSAVDPTK.D K.MYYSAVDPTKDIFTGLIGPMK.I K.DIFTGLIGPMK.I R.MFTTAPDQVDK.E R.MFTTAPDQVDKEDEDFQESNK.M R.QSEDSTFYLGER.T R.QYTDSTFR.V R.KAEEEHLGILGPQLHADVGDK.V K.DLYSGLIGPLIVCR.R K.VNKDDEEFIESNK.M	20.6 (19)	Copper-binding glycoprotein, ferroxidase activity, iron transport

2	Q14624	inter-alpha trypsin inhibitor heavy chain H4	ITIH4_HUMAN	2.11	103357	6.51	74	10	98 - 112 152 - 163 214 - 225 299 - 308 428 - 439 500 - 513 633 - 645 657 - 670 815 - 827 831 - 843	K.AEAQAQYSAAVAK.G R.RLGVYELLLK.V R.FKPTLSQQQK.S K.ILDDLSPR.D K.LALDNGGLAR.R R.GPDVLTATVSGK.L K.IPKPEASFSPR.R R.MNFRPGVLSSR.L K.ETLFSVMPGLK.M K.TGLLLLSDPDK.V	11.5 (10)	Serine-type endopeptidase inhibitor, acute-phase response, hyaluronan metabolic process

3	P0C0L4	complement C4-A precursor (Two spots identified for this protein)	CO4A_HUMAN	2.61	192771	6.66	192, 93	8, 4	756 - 776 916 - 930 979 - 1006 1042 - 1052 1084 - 1100 1291 - 1301 1340 - 1350 1352 - 1366	R.ALEILQEEDLIDEDDIPVR.S R.GSFEFPVGDAVSK.V R.VTASDPLDTLGSEGALSPGGVASLLR.L K.DHAVDLIQK.G K.VLSLAQEQVGGSPEK.L R.QGSFQGGFR.S K.SHALQLNNR.Q R.GLEEELQFSLGSK.I	6.5 (8)	Activation of the classical pathway of the complement system

4	P01031	complement C5 precursor	CO5_HUMAN	1.59	188305	6.11	154	6	78 - 91 308 - 322 353 - 373 436 - 450 495 - 507 622 - 630	K.FQNSAILTIQPK.Q K.ELSYYSLEDLNNK.Y K.LNLVATPLFLKPGIPYPIK.V K.TDAPDLPEENQAR.E K.ITHYNYLILSK.G R.VFQFLEK.S	4.5 (6)	Complement cascade through to formation of membrane attack complex (MAC), inflammatory response

5	P02748	complement component C9 precursor	CO9_HUMAN	2.38	63173	5.43	196	7	65 - 77 144 - 155 145 - 155 231 - 243 472 - 484 496 - 509 533 - 544	R.SIEVFGQFNGK.R R.DRVVEESELAR.T R.VVEESELAR.T K.TSNFNAAISLK.F K.LSPIYNLVPVK.M R.AIEDYINEFSVR.K K.FEGIACEISK.Q	11.8 (7)	Pore-forming subunit of the MAC that plays a key role in the innate and adaptive immune response

6	P01042	kininogen-1 (Two spots identified for this protein)	KNG1_HUMAN	1.72	71957	6.34	37, 187	6, 9	43 - 59 64 - 76 101 - 114 187 - 197 208 - 220 240 - 255 254 - 263 316 - 325 380 - 390	K.YNSQNQSNNQFVLYR.I K.TVGSDTFYSFK.Y K.AATGECTATVGK.R R.QVVAGLNFR.I K.ENFLFLTPDCK.S R.IASFSQNCDIYPGK.D K.DFVQPPTK.I K.YFIDFVAR.E K.RPPGFSPFR.S	15.1 (9)	Role in blood coagulation, inhibitor of thiol proteases, inflammatory response, diuresis and natriuresis, smooth muscle contraction, negative regulation of cell adhesion

7	P09871	complement C1s subcomponent precursor (Three spots identified for this protein)	C1S_HUMAN	4.45	76684	4.86	190, 66	8, 5, 6	86 - 106 264 - 281 314 - 332 369 - 384 481 - 497 515 - 524 523 - 535 629 - 645 677 - 688	R.SSNNPHSPIVEEFQVPYNK.L K.SNALDIIFQTDLTGQK.K R.DVVQITCLDGFEVVEGR.V K.VEDPESTLFGSVIR.Y R.EPTMYVGSTSVQTSR.L K.LLEVPEGR.T R.TNFDNDIALVR.L K.GDSGGAFAVQDPNDK.T K.TMQENSTPRED.-	18.3 (9)	Serine-type endopeptidase involved in activation of classical pathway of the complement system

The table shows those proteins that are differentially expressed between DS and Ctl pregnancies in the second trimester in the pH range 4.5 - 5.5. All results displayed here are from ESI Q-TOF MS/MS analysis. Identified proteins are given with UniProt accession number, average fold up-regulation in DS samples compared to Ctl samples (from DeCyder analysis; comparable to Progenesis), theoretical molecular weight and p*I *(calculated using the Compute pI/mW function available on the ExPASy website, full amino acid sequence used including signal peptides), search score and number of peptides used for identification. The sequences of the peptides used for the identification of the protein spots (M indicates methionine oxidation) and their position within the protein sequence are shown; also the function of the protein. Coverage indicates the percentage of the residues in each protein sequence that was identified. Score is -10*Log(P), where P is the probability that the observed match is a random event. Scores > 46 indicate identity or extensive homology at the p < 0.05 level.

#### 4.5 - 5.5 pH range

Two sets of 7 paired samples were tested for the 4.5 - 5.5 pH range for the first trimester, giving a total of 14 gestationally age-matched pairs of Ctl and DS samples. With the first 7 pairs of samples, there were up to 13 protein spots differentially expressed between DS and Ctl samples, depending on the software analysis package used. Only two protein spots matched between the analyses with Progenesis Same Spots and DeCyder v6.5, highlighting the differences in algorithms used. With the second set of 7 pairs of DS and Ctl matched samples, none of the initial spots could be confirmed as differentially expressed. It was concluded that there were no differentially expressed proteins in this pH range for first trimester samples.

**Figure 1:**
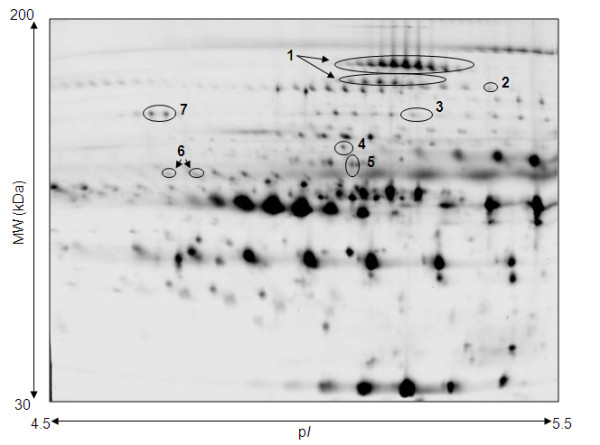
**Representative 2D DIGE gel of second trimester maternal plasma in pH 4.5 - 5.5 range**. Image shown is from Cy2 signal. Spots numbered are those shown to be significantly differentially expressed in DS samples by more than 1.5 fold (p < 0.05) compared with controls. Eight gels were run (DS [n = 8] and Ctl [n = 8]). See Table 1 for mass spectrometry-based identifications. 1 = ceruloplasmin, 2 = inter-alpha-trypsin inhibitor heavy chain H4, 3 = complement C4-A precursor, 4 = complement C5 precursor, 5 = complement component C9 precursor, 6 = kininogen-1, 7 = complement C1s subcomponent precursor

**Figure 2:**
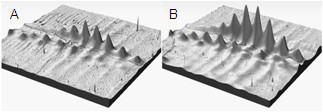
**3D representation of volume of ceruloplasmin protein spots on 2D DIGE gel**. Representation is for pH 4.5 - 5.5 range for second trimester samples. A) Ctl protein spots; B) DS protein spots.

For the second trimester, eight pairs of age-matched DS and Ctl samples were subjected to 2D DIGE over the pH 4.5 - 5.5 range. Figure 1 shows a representative 2D gel image with the differentially expressed proteins between the two groups highlighted, whilst Figure 2 gives a 3D representation of the ceruloplasmin protein spots. Table 1 summarizes the spot number, symbol, Swiss-Prot accession number, theoretical p*I*, molecular weight, score, number of peptides used for identification and protein coverage for the differentially expressed proteins. The proteins shown are those that matched from the two software analyses. Seven proteins showed up-regulation in DS *versus *Ctl samples - ceruloplasmin, inter-alpha-trypsin inhibitor heavy chain H4, complement proteins C1s subcomponent, C4-A, C5, and C9 and kininogen 1.

#### 5.3 - 6.5 pH range

14 pairs of samples from the first trimester were analysed for the pH range 5.3 - 6.5. No protein spots were found to be differentially expressed between DS samples and Ctl samples with more than 1.5 fold up- or down-regulation and both software packages. This was also the case for the second trimester samples.

#### 6 - 9 pH range

All 14 pairs of samples from the first trimester were analysed by 2D DIGE for the pH 6 - 9 range. No protein spots were altered by ≥1.5 fold in DS samples compared to Ctl samples by both software packages.

**Figure 3:**
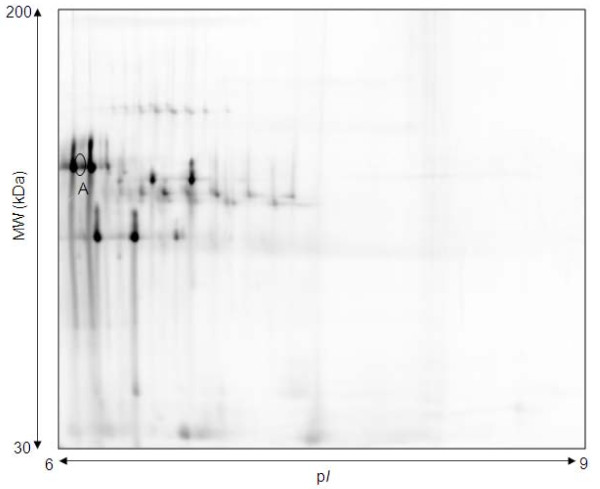
**Representative 2D DIGE gel of second trimester maternal plasma in pH 6 - 9 range**. Image shown from Cy2 signal. Spot A is significantly up-regulated in DS samples by more than 1.5 fold (p < 0.05) compared with Ctl samples. Protein was unable to be identified. Nine gels were run (DS [n = 9] and Ctl [n = 9]).

2D DIGE data from 9 pairs of DS and Ctl samples from the second trimester led to the conclusion that one protein spot was differentially expressed between DS and Ctl groups (marked A in Figure 3). However, identification of this protein proved elusive, mainly hampered by an excess of serotransferrin in the same region.

### Confirmatory Experiments

**Figure 4:**
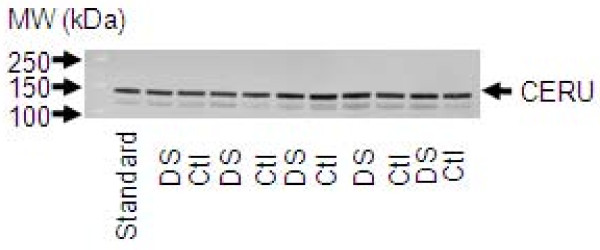
**Representative 1D Western blot for anti-ceruloplasmin expression**. Second trimester maternal plasma samples were used (in total, DS [n = 10] and Ctl [n = 10]). Equal amounts of plasma from all samples were separated by gel electrophoresis and blotted with the appropriate dilution of antibody (refer to Materials and Methods). A standard control sample (labelled as 'Standard') was selected and run on every blot to use as a calibration. No significant difference was seen between DS and Ctl levels of ceruloplasmin (CERU; running on 1D gel at 132 kDa).

Previous studies [11,17] have shown an increase in the levels of ceruloplasmin in DS samples compared to Ctl samples in first and second trimesters. In our 2D DIGE experiments, we confirmed this change in the second but not the first trimester. We assessed whether the difference in ceruloplasmin protein expression between DS and Ctl samples could be detectable with an anti-ceruloplasmin mAb and conventional 1D Western blotting. After using a CyDye-conjugated secondary antibody and ImageQuant software v5.2, we detected no statistically significant difference between the expression levels of ceruloplasmin in the two sample groups (Figure 4).

## Discussion

At present, diagnosis of DS involves the invasive techniques of amniocentesis and CVS. There have been significant advances in NIPD for DS in recent years including the assessment of free fetal DNA in maternal plasma using next generation sequencing [22-26]. However, this is an expensive technique so there is still scope for the use of new plasma biomarkers for prenatal screening.

This study focused in more depth on the depleted maternal plasma proteome for DS biomarkers than previous studies by investigating expanded pH ranges for 2D DIGE. This approach achieves better separation and resolution of proteins, giving greater analysis of differential expression. Broader range pH gels have overlapping protein separations making accurate quantification difficult and obscuring low abundance proteins. In this study, no significant differential protein expression was observed in the first trimester. In the second trimester, protein changes detected in maternal plasma in the pH 4.5 - 5.5 range included increased levels of ceruloplasmin, inter-alpha-trypsin inhibitor heavy chain H4, complement proteins C1s subcomponent, C4-A, C5, and C9 and kininogen 1 in DS samples. Some of these changes correspond to those found by other groups [11,17,18] (ceruloplasmin, inter-alpha-trypsin inhibitor heavy chain H4, complement component C9 and kininogen 1). One of the greatest changes in the current study was in ceruloplasmin protein expression - 3.69 fold up-regulated in DS - a larger fold change than seen by other groups. However, when ceruloplasmin levels were specifically assessed by Western blotting, no significant difference could be seen between samples. This was most likely due to the fact that Western blotting is only semi-quantitative. The epitope of ceruloplasmin that the primary antibody was designed against is unknown and hence changes between DS and Ctl samples may be detected by the use of a different primary antibody. Ceruloplasmin is seen as two chains of spots with 2D DIGE and the changes detected may be a change in post-translational modification of the protein as well as total level of protein. The more stringent 2D DIGE approach of the current study did not corroborate many of the previous findings by other groups [11,17], who have performed 2DE using a broader pH range. Differences in method (including study size) and sample preparation (including collection, processing and storage) may contribute to discrepancies. We have compared all known mass spectrometry-based 2DE based proteomic identifications of DS plasma markers (see Additional file 1, Table S1).

The potential biomarkers highlighted here are involved with fetal growth and development. Ceruloplasmin transports copper and ceruloplasmin levels increase during pregnancy. The impaired transport of copper has been associated with poor pregnancy outcome [27,28]. Increased ceruloplasmin levels have been detected in postmortem brain samples from patients with a variety of neurodegenerative diseases [29], implicating a possible change in oxidative stress in regions of the brain. However, Tórsdóttir *et al. *[30] showed there was no change in ceruloplasmin oxidative activity that could be correlated with dementia in DS. In a recent study using a redox proteomics approach, ceruloplasmin was shown to be increasingly carbonylated in DS amniotic fluid samples [20]. Inter-alpha-trypsin inhibitor heavy chain H4 levels were also shown to be increased in DS in this study. This is a plasma glycoprotein, which is primarily expressed in the liver and can act as an acute-phase protein in several species. It acts as a substrate for plasma peptidase kallikrein [31] but its exact role *in vivo *remains unclear. Four of the proteins shown to be up-regulated in DS samples belong to the complement system. Perluigi *et al. *[20] suggest that increased levels of the oxidized form of complement C9 may lead to a loss of biological function, altering its role in the acute-phase response protecting the developing fetus. Kininogen 1 was shown to be up-regulated in DS samples in this study. This protein has a role to play in the blood coagulation system, as well as the kinin-kallikrein system. Kininogen was also found to be oxidized in DS amniotic fluid [20].

In women bearing DS fetuses placental development is often abnormal [32]. The process of cytotrophoblast fusion to form syncytiotrophoblast does not occur properly [33]. Defective placental development may result in an elevated level of placental debris [12]. Several of the peptides identified in the current study are likely to be fragments of the larger full-length proteins, given that the molecular weight and p*I *of the intact proteins (see Table 1) do not always agree with the location of the protein spots on the 2D gels (see Figure 1). This implies enzymatic activity in the plasma samples resulting in the cleavage of the proteins.

The genes encoding the proteins that have been shown to be differentially expressed between DS and Ctl samples in this study are located on chromosomes other than chromosome 21. This implies that proteins encoded by genes located on chromosome 21 (such as transcription factors) are influencing the expression of genes on other chromosomes. The current study did not detect changes in expression in the current protein screening markers (PAPP-A, AFP, hCG, inhibin A) between DS and Ctl samples. This was also the case in other 2DE studies [11,17] but two groups [12,19] detected an increase in beta hCG in DS samples, suggesting that there is an increase in sensitivity in iTRAQ analysis and 2D LC-MS/MS analysis *versus *a 2D gel approach. Another reason for the lack of detection of the current screening markers is that some proteins are more amenable to being detected by mass spectrometry than others.

Proteomic methods have also been applied for the identification of biomarkers in other fetal aneuploidies - Turner Syndrome (XO karyotype) [34,35] and Klinefelter syndrome (XXY karyotype) [36]. Most of the biomarkers identified in these studies also show changes in expression in DS, indicating they are not specific to an extra copy of chromosome 21 (this paper; [11,12,17]).

A recent study has used integrative data mining as an approach to try to identify biomarkers for DS screening [37]. Also bead-based multiplexed immunoassays have identified new DS biomarkers [38] and epidermal growth factor was confirmed as a potential biomarker following a validation study [39]. Lopez *et al. *performed selective reaction monitoring following mass spectrometry biomarker discovery, thus developing high throughput robust assays for DS detection [40].

## Conclusion

This study has shown the up-regulation of seven protein biomarkers in the maternal plasma of DS pregnancies compared to Ctl pregnancies in the second trimester of pregnancy, with overlap between these identified biomarkers and previous studies. These markers need to be assessed in a larger cohort of pregnancies and in the wider population before they could potentially be used as better screening markers and/or for diagnosis. Given such an extensive study of different pH ranges, to find only seven altered proteins is surprising. However, it must be appreciated that gel-based approaches for protein identification are limited. Gel-free-based approaches may be more informative. There is scope for improvement in the current screening protocols and further work needs to be done to detect and diagnose DS in the first trimester non-invasively. The ultimate goal remains the introduction of NIPD tests that are rapid, inexpensive, automated and accurate.

## Methods

### Materials

Maternal peripheral blood (in EDTA tubes) was taken with informed consent from pregnant women (majority of women of Caucasian background) attending the Fetal Medicine Unit, University College Hospital, London, U.K. for invasive diagnostic testing for a range of clinical indications as part of the SAFE Framework 6 Network of Excellence study [9]. Fourteen blood samples were obtained from normal pregnancies (Ctl) and 14 from DS affected pregnancies in the 10-14 week (wk) gestation period (first trimester) (average difference in gestational age between matched samples 3.5 days). The DS status was determined by karyotype analysis from CVS or amniotic fluid at University College Hospital, London. A similar number of samples were obtained in the 14-28 wk gestation period (second trimester) (average difference in gestational age between matched samples 10.5 days). Samples were analysed independently (hence providing fourteen potential biological replicates per trimester, see Additional file 2, Table S2 to see how Ctl and DS samples were matched as pairs). Maternal age ranged from 21-45 years for Ctl pregnancies and from 19-42 years for DS affected pregnancies (average maternal age Ctl pregnancies first trimester 35.4 years, average maternal age DS affected pregnancies first trimester 36.3 years, average maternal age Ctl pregnancies second trimester 35 years, average maternal age DS affected pregnancies second trimester 36.1 years, no significant difference between Ctl and DS groups for either trimester). Plasma was obtained by centrifuging the blood samples at 1,500 g for 10 minutes (min) and further separated by centrifuging at 16,000 g for 15 min. Protease inhibitor cocktail (Sigma-Aldrich, Dorset, U.K.) was added and fractions were stored at -80°C prior to analysis. 55 of 56 samples were processed within 24 hours (hrs) of collection, with 40 of these being processed within 8 hrs of collection (no significant difference between processing of Ctl and DS samples). Collection of samples took place from January 2006 to June 2007 and samples were analysed by 2D DIGE during this period and up to March 2008.

### Methods

#### Sample Preparation

Plasma samples were depleted for albumin and IgG using the Qproteome Albumin/IgG Depletion Kit (QIAGEN, Crawley, U.K.). Depleted protein was eluted into 50 mM ammonium bicarbonate buffer pH 7.8 and assayed for protein concentration using the Bicconinic acid kit (Sigma-Aldrich, Dorset, U.K.) or samples were eluted into 50 mM Tris Cl; 4% *w/v *CHAPS; 200 mM urea, pH 7.5 and cleaned up for 2DE using the 2D Clean-Up Kit (GE Healthcare, Little Chalfont, U.K.). The latter samples were assayed for protein concentration using the NanoOrange^® ^Protein Quantitation Kit (Invitrogen, Paisley, U.K,). Protein was aliquoted to required amounts, frozen or freeze dried.

#### 2D DIGE and Image analysis

Depleted plasma protein samples (30 μg) were re-suspended in labelling buffer (7 M Urea, 2 M Thiourea, 2% *w/v *CHAPS, 10-20 mM Tris) and labelled with CyDye DIGE Fluor minimal dyes (GE Healthcare, Little Chalfont, U.K.) (reconstituted in fresh 99.8% anhydrous DMF) at a concentration of 400 pmol of dye/50 μg of protein. Ctl samples were labelled with Cy3 and DS samples were labelled with Cy5 or vice versa and a standard pool consisting of protein from all samples was labelled with Cy2. Labelling reactions were halted with 10 μM lysine (Sigma-Aldrich, Dorset, U.K.). All three labelled samples were combined and resolved in one gel. CyDye labelling of Ctl and DS samples was mixed equally. Combined samples were added to IPG strip rehydration buffer (7 M Urea, 2 M Thiourea, 2% *w/v *CHAPS, 50 mM DTE and 0.5-1% *v/v *relevant IPG buffer) and applied to either pH 4.5 - 5.5, pH 5.3 - 6.5 or pH 6 - 9 24 cm immobiline drystrips (GE Healthcare, Little Chalfont, U.K.), covered with mineral oil and left at room temperature for 12-24 hrs. Drystrips were run on an IPG Multiphor (GE Healthcare, Little Chalfont, U.K.) for a total of 90-100,000 Vh with a 50 μA/drystrip limit. Strips were incubated for 15 min each in 10 mg/mL DTE equilibration buffer and then 25 mg/mL iodoacetamide equilibration buffer and resolved on 12% acrylamide gels, using piperidinediacrylamide (Bio-Rad, Hemel Hempstead, U.K.) as a crosslinker. Gels were run in a Hoefer DALT tank using the Ettan DALT electrophoresis system (GE Healthcare, Little Chalfont, U.K.). Gels were scanned on a Typhoon 9400 Variable Mode Imager (GE Healthcare, Little Chalfont, U.K.) at a resolution of 100 μm. 2D gel images were analysed for differentially expressed spots using Progenesis SameSpots software version 3.1 (Nonlinear Dynamics Ltd, Newcastle upon Tyne, U.K.) or DeCyder v6.5 (GE Healthcare, Little Chalfont, U.K.), following protein spots being detected, aligned and matched between gels. Normalized protein spots in the Cy5 and Cy3 channels were compared to the internal standard (Cy2) to generate a ratio of relative amount. Ctl and DS samples were compared using Student's t test analysis. A threshold level was set of 1.5 fold up- or down-regulation, at p < 0.05 level.

#### MALDI-TOF MS and ESI Q-TOF MS/MS analyses

12% bis acrylamide preparative 2D gels were run with 1.5 mg of pooled depleted freeze dried plasma protein. Gels were either colloidal Coomassie blue (Sigma-Aldrich, Dorset, U.K.) or silver stained and spots trypsin digested for MALDI-TOF analysis according to previously described methods [41,42]. Following tryptic digestion, peptides were extracted in 50% *v/v *ACN/0.1% *v/v *TFA and spotted with 1:1 ratio of 10 mg/mL alpha-cyano-4-hydroxycinnamic acid in 50:50 *v/v *methanol/ACN. Samples were analysed using a MALDI-TOF mass spectrometer (Waters-Micromass, Elstree, U.K.), as previously described [42].

Some samples were analysed using a Waters Q-TOF Micro mass spectrometer (Waters-Micromass, Elstree, U.K.) to obtain nanoelectrospray ionization tandem mass spectra. Samples were separated on a Dionex Ultimate 1 nanoflow-LC system (Dionex, Camberley, U.K.) using a 15 cm × 0.75 mm Acclaim PepMap100 C_18 _3 μm column with a flow rate of 200 nL/min. Solvent A was 5% *v/v *ACN/95% *v/v *0.1% *v/v *aqueous formic acid and solvent B was 80% *v/v *ACN/20% *v/v *0.1% *v/v *aqueous formic acid and a gradient time of 60 min was used. MS/MS spectra were acquired in data-dependent mode, processed using ProteinLynx Global Server v.2.1 software and then protein identities confirmed using MASCOT (SwissProt database version 55.4 [385721 sequences] to version 56.0 [392667 sequences]) (Matrix Science Ltd., London, U.K.). Search criteria were: peptide tolerance of 100 parts per million (ppm); fragment tolerance of 0.1Da; two trypsin missed cleavages per peptide; fixed carbamidomethylation of cysteine and variable oxidation of methionine modifications. Only identities from MASCOT with three or more peptides were considered.

As several peptides from a protein were identified from different Q-TOF analyses, Protein Coverage Summarizer software [43] was used to determine the percentage of the residues in each protein sequence that was identified (coverage column in Table 1).

#### Western Blot analysis

Plasma samples were diluted in PBS and the equivalent of 0.75 μL of plasma were separated by 4-12% gradient reducing SDS-PAGE. A standard control sample was run on every blot to use as a calibration. Ten different DS samples and 10 different Ctl samples were run and proteins were blotted onto Hybond-LFP PVDF membrane (GE Healthcare, Little Chalfont, U.K.). Blots were blocked for 1 hr in 2% *w/v *ECL Advance Block (GE Healthcare, Little Chalfont, U.K.) in PBS/1% *v/v *Tween 20. Membranes were incubated overnight at 4°C with 1:1000 dilution of mouse mAb to ceruloplasmin (SC-69767; Santa Cruz, Germany) (in blocking buffer). Blots were washed 6 x10 min with 0.1% *v/v *Tween 20 in PBS before being incubated for 1 hr with anti-mouse Cy3 conjugated secondary antibody (GE Healthcare, Little Chalfont, U.K.) at a 1:2500 dilution. Blots were washed and dried overnight in the dark then scanned for Cy3 dye fluorescence using a Typhoon 9400 Variable Mode Imager. ImageQuant software v5.2 (Molecular Dynamics, U.K.) was used to measure volume, background intensity was subtracted, every blot was run in triplicate and intensity was calibrated and averaged for each sample. Statistical analysis was performed using Microsoft Excel 2003 software. Student's t-test was used to test for significance between the protein levels in Ctl *versus *DS maternal plasma samples.

## Abbreviations

2D DIGE: two-dimensional difference gel electrophoresis; 2DE: two-dimensional gel electrophoresis; AFP: alpha-fetoprotein; CVS: chorionic villus sampling; DS: Down Syndrome; ESI Q-TOF MS/MS: electro-spray ionisation quadrupole time-of-flight tandem mass spectrometry; hCG: human chorionic gonadotropin; hrs: hours; MAC: membrane attack complex; min: minutes; NIPD: non-invasive prenatal diagnosis; PAPP-A: pregnancy-associated plasma protein A; ppm: parts per million; TTP: time to process; uE3: unconjugated estriol; wks: weeks.

## Competing interests

The authors declare that they have no competing interests.

## Authors' contributions

WEH participated in the design of the study, carried out the sample preparation, 2D DIGE and image analysis, some mass spectrometry analysis and helped draft the manuscript. TEM participated in the design of the study, carried out the sample preparation, 2D DIGE and image analysis, Western blotting analysis and helped draft the manuscript. DW carried out the sample preparation. AW participated in the interpretation of data. JH carried out the sample collection. KM carried out mass spectrometry analysis and participated in the design of the study. NDA conceived of the study and participated in its design and coordination. All authors read and approved the final manuscript.

## Supplementary Material

Additional file 1 **Potential biomarkers for DS identified by mass spectrometry-based 2DE-based proteomic studies**. In Table S1, we have compared all known mass spectrometry-based 2DE-based proteomic identifications of DS plasma markers. Click here for file

Additional file 2 **Clinical and demographic data for maternal peripheral blood samples**. In Table S2, all relevant information about the Ctl/DS sample pairs for the first and second trimesters is detailed. Click here for file
