# Supplementary material for: 2D DIGE analysis of maternal plasma for potential biomarkers of Down Syndrome
Source: Proteome Sci. 2011 Sep 19;9:56. doi: 10.1186/1477-5956-9-56 (PMC3189872; doi:10.1186/1477-5956-9-56)
Supplement: Additional file 1 — Potential biomarkers for DS identified by mass spectrometry-based 2DE-based proteomic studies. In Table S1, we have compared all known mass spectrometry-based 2DE-based proteomic identifications of DS plasma markers. [file 1477-5956-9-56-S1.PDF]

Table S1. Potential biomarkers for DS identified by mass spectrometry-based 2DE-based proteomic studies

					1st trimester			2nd trimester		
**Protein**	**Abbreviated name (all listed are _HUMAN)**	**Accession Code**	**1st trimester**	**2nd trimester**	**MS methods**	**Sample source**	**Reference**	**MS methods**	**Sample source**	**Reference**
afamin	AFAM	P43652	up	up	2-D DIGE - MS/MS	serum	[17]	2-D DIGE - MS/MS; 2-DE - MALDI-TOF/MS	serum; plasma	[17]; [11]
alpha-1-acid glycoprotein 1	A1AG1	P02763	up		2-D DIGE - MS/MS	serum	[17]			
alpha-1-antitrypsin	A1AT	P01009		up				2-DE - MALDI-TOF/MS	plasma	[11]
alpha-1-microglobulin	AMBP	P02760		up				2-DE - MALDI-	amniotic fluid;	[16]; [17]; [11]

								TOF/nano-ESI-MS/MS; 2-D DIGE -MS/MS; 2-DE -MALDI-TOF/MS	serum; plasma	
alpha-2-HS-glycoprotein	FETUA	P02765	up	up	2-D DIGE -MS/MS	serum	[17]	2-D DIGE -MS/MS	serum	[17]
alpha-2-macroglobulin	A2MG	P01023		up				2-D DIGE -MS/MS	serum	[17]
apolipoprotein A-I	APOA1	P02647	up		2-D DIGE -MS/MS	serum	[17]			
apolipoprotein A-IV	APOA4	P06727	up		2-D DIGE -MS/MS	serum	[17]			
apolipoprotein C-II	APOC2	P02655	down	down	2-D DIGE -MS/MS	serum	[17]	2-D DIGE -MS/MS	serum	[17]

apolipoprotein E	APOE	P02649	up	up	2-D DIGE - MS/MS	serum	[17]	2-DE - MALDI-TOF/MS	plasma	[11]
basement membrane-specific heparin sulfate proteoglycan core protein	PGBM	P98160		up				2-DE - MALDI-TOF/nano-ESI-MS/MS	amniotic fluid	[16]
beta-2-glycoprotein 1	APOH	P02749	up	up	2-D DIGE - MS/MS	serum	[17]	2-D DIGE - MS/MS	serum	[17]
ceruloplasmin	CERU	P00450	up	up	2-D DIGE - MS/MS	serum	[17]	2-D DIGE - MALDI-TOF MS/ESI Q-TOF MS/MS; 2-D DIGE - MS/MS; 2-	plasma; serum	this paper; [17]; [11]

								DE - MALDI-TOF/MS		
clusterin	CLUS	P10909	up	down	2-D DIGE - MS/MS	serum	[17]	2-DE - MALDI-TOF/MS	plasma	[11]
collagen alpha 1 (I) chain	CO1A1	P02452		up				2-DE - MALDI-TOF/nano-ESI-MS/MS	amniotic fluid	[16]
collagen alpha 1 (III) chain	CO3A1	P02461		up				2-DE - MALDI-TOF/nano-ESI-MS/MS	amniotic fluid	[16]
collagen alpha 1 (V) chain d	CO5A1	P20908		up				2-DE - MALDI-	amniotic fluid	[16]

								TOF/nano-ESI-MS/MS		
complement C1S component	C1s	P09871	up	up	2-D DIGE - MS/MS	serum	[17]	2-D DIGE - MALDI-TOF MS/ESI Q-TOF MS/MS	plasma	this paper
complement C3	CO3	P01024		up				2-D DIGE - MS/MS	serum	[17]
complement C4-A precursor	CO4A	P0C0L4	up	up	2-D DIGE - MS/MS	serum	[17]	2-D DIGE - MALDI-TOF MS/ESI Q-TOF MS/MS	plasma	this paper
complement C5	CO5	P01031		up				2-D DIGE -	plasma	this paper

								MALDI-TOF MS/ESI Q-TOF MS/MS		
complement component C9	CO9	P02748		up				2-D DIGE - MALDI-TOF MS/ESI Q-TOF MS/MS	plasma	this paper
complement factor H	CFAH	P08603	up	up	2-D DIGE - MS/MS	serum	[17]	2-D DIGE - MS/MS	serum	[17]
ficolin 3	FCN3	O75636	down		2-D DIGE - MS/MS	serum	[17]			
gelsolin	GSN	P06396		up				2-D DIGE - MS/MS	serum	[17]
histidine-rich	HRG	P04196		up				2-DE -	plasma	[11]

glycoprotein								MALDI-TOF/MS		
inter-alpha-trypsin inhibitor heavy chain H2	ITIH2	P19823	up	up	2-D DIGE - MS/MS	serum	[17]	2-D DIGE - MS/MS	serum	[17]
inter-alpha-trypsin inhibitor heavy chain H4	ITIH4	Q14624	up	up	2-D DIGE - MS/MS	serum	[17]	2-D DIGE - MALDI-TOF MS/ESI Q-TOF MS/MS; 2-D DIGE - MS/MS	plasma; serum	this paper; [17]
kininogen 1	KNG1	P01042		up				2-D DIGE - MALDI-TOF MS/ESI Q-TOF	plasma	this paper

								MS/MS		
pigment epithelium-derived factor	PEDF	P36955	up	up	2-D DIGE - MS/MS	serum	[17]	2-D DIGE - MS/MS	serum	[17]
protein IBP-1	IBP-1	P08833		down				2-DE - MALDI-TOF/nano-ESI-MS/MS	amniotic fluid	[16]
serum amyloid A	SAA	P04278		up				2-D DIGE - MS/MS	serum	[17]
serum amyloid P-component	SAMP	P02743		up				2-DE - MALDI-TOF/MS	plasma	[11]
sex hormone-binding globulin	SHBG	P04278	up		2-D DIGE - MS/MS	Serum	[17]			
splicing factor arginine/serine-rich 4	SFRS4	Q08170		up (only present in ds samples)				2-DE - MALDI-TOF/nano-	amniotic fluid	[16]

								ESI- MS/MS		
tetranectin	TETN	P05452		up				2-D DIGE - MS/MS	serum	[17]
transthyretin	TTHY	P02766	up	up	2-D DIGE - MS/MS	serum	[17]	2-D DIGE - MS/MS; 2-DE - MALDI-TOF/MS	serum; plasma	[17]; [11]

Identified biomarkers are given with SwissProt accession number, regulation in DS samples compared to Ctl samples (up = up-regulation in DS samples; down = down-regulation in DS samples compared to Ctl samples), MS methods, sample source and reference.

Summary of MS methods:

[16]: 2-DE – MALDI-TOF/nano-ESI-MS/MS

[17]: 2-D DIGE – MS/MS

[11]: 2-DE – MALDI-TOF/MS

This paper: 2-D DIGE – MALDI-TOF MS/ESI Q-TOF MS/MS
