# Supplementary material for: 2D DIGE analysis of maternal plasma for potential biomarkers of Down Syndrome
Source: Proteome Sci. 2011 Sep 19;9:56. doi: 10.1186/1477-5956-9-56 (PMC3189872; doi:10.1186/1477-5956-9-56)
Supplement: Additional file 2 — Clinical and demographic data for maternal peripheral blood samples. In Table S2, all relevant information about the Ctl/DS sample pairs for the first and second trimesters is detailed. [file 1477-5956-9-56-S2.PDF]

Table S2. Clinical and demographic data for maternal peripheral blood samples

Trimester	Sample Set	Sample Type	Gestational Age	Fetal Sex	Maternal Age	Maternal Weight (kg)	Maternal Ethnicity	TTP (hrs)
1st	1	DS	10+6	female	40	55	Caucasian	3
1st	1	Ctl	11+3	female	45	58	Caucasian	2.75
1st	2	DS	12+0	male	40	55	Caucasian	6.25
1st	2	Ctl	11+5		32		Caucasian	7.5
1st	3	DS	12+0	male	41	108	Caucasian	7
1st	3	Ctl	11+4	male	23	50	Caucasian	5
1st	4	DS	12+0	male	42	60	non-Caucasian	6.5
1st	4	Ctl	13+0	male	35	50		4.5
1st	5	DS	12+1	male	42	66	Caucasian	<6
1st	5	Ctl	12+4	female	39	90	Caucasian	5
1st	6	DS	12+3	male	19		Caucasian	2
1st	6	Ctl	13+4		21	49.5	Caucasian	1.5
1st	7	DS	12+5	female	34	75	Caucasian	22.5
1st	7	Ctl	13+1	female	39	67	non-Caucasian	23
1st	8	DS	13+0	female		70	Caucasian	4.75
1st	8	Ctl	12+5	male	38	75	non-Caucasian	4.25
1st	9	DS	13+0	male	38	60	Caucasian	3.5
1st	9	Ctl	13+1	male	37	55		5
1st	10	DS	13+0	male	40	55	Caucasian	5
1st	10	Ctl	13+1		35		Caucasian	4.75
1st	11	DS	13+1	male	38	60	Caucasian	
1st	11	Ctl	12+3	female	39	76	Caucasian	2
1st	12	DS	13+1	female	38	70	Caucasian	1
1st	12	Ctl	13+1		38		non-Caucasian	3.5

1st	13	DS	13+6	male	28	55	Caucasian	4.5
1st	13	Ctl	13+5	male	39	76	non-Caucasian	2
1st	14	DS	13+0	female	32	50	Caucasian	4.5
1st	14	Ctl	13+5	male	36	70	Caucasian	7.5
2nd	15	DS	14+0	female	38	60	Caucasian	
2nd	15	Ctl	14+4	female	33	82	Caucasian	22.25
2nd	16	DS	15+3	male	36		non-Caucasian	22.5
2nd	16	Ctl	14+0	male	45		Caucasian	28.5
2nd	17	DS	16+1	female	37	51	non-Caucasian	24
2nd	17	Ctl	16+0	female	35	60	Caucasian	20
2nd	18	DS	16+5	male	42	60	Caucasian	
2nd	18	Ctl	17+1	male	39	82	Caucasian	2.75
2nd	19	DS	17+6	male	31	73	Caucasian	2
2nd	19	Ctl	18+2	female	32		Caucasian	6
2nd	20	DS	20+4	female	31	55	Caucasian	22.5
2nd	20	Ctl	21+0	male	31	90	Caucasian	23
2nd	21	DS	21+1	male	33	50	Caucasian	24
2nd	21	Ctl	19+0	female	39	80	Caucasian	20
2nd	22	DS	21+2	female	41	67	Caucasian	2
2nd	22	Ctl	19+1	female	35	54	non-Caucasian	
2nd	23	DS	23+1	male	39	65	Caucasian	2.5
2nd	23	Ctl	22+4	male	36	48	Caucasian	7
2nd	24	DS	23+5		40	68	Caucasian	1.5
2nd	24	Ctl	20+6	female	36	80		3.75
2nd	25	DS	24+6		37	58	non-Caucasian	2.5
2nd	25	Ctl	21+6	female	42	87	Caucasian	6.5
2nd	26	DS	24+6	male	38	115	non-Caucasian	3.5
2nd	26	Ctl	23+0	male	28		Caucasian	2.5
2nd	27	DS	25+5	female	24	75	Caucasian	1.5

2nd	27	Ctl	27+5	male	25	67	Caucasian	7
2nd	28	DS	30+0	male	38	90	non-Caucasian	19.5
2nd	28	Ctl	32+1	male	34	110	Caucasian	5.5

Sample Set indicates which DS and Ctl sample were paired together for 2D DIGE gels.

TTP indicates time to process for the samples i.e. the time between the sample being taken and the time when it was processed and stored at -80°C.
